# Altered expression of fibroblast activation protein-α (FAP) in colorectal adenoma-carcinoma sequence and in lymph node and liver metastases

**DOI:** 10.18632/aging.103261

**Published:** 2020-05-19

**Authors:** Jon Danel Solano-Iturri, Maider Beitia, Peio Errarte, Julio Calvete-Candenas, María C. Etxezarraga, Alberto Loizate, Enrique Echevarria, Iker Badiola, Gorka Larrinaga

**Affiliations:** 1 Department of Anatomic Pathology, Cruces University Hospital, University of the Basque Country (UPV/EHU), Barakaldo, Spain; 2 BioCruces Health Research Institute, Barakaldo, Spain; 3 Department of Nursing, Faculty of Medicine and Nursing, University of the Basque Country (UPV/EHU), Leioa, Spain; 4 Department of Physiology, Faculty of Medicine and Nursing, University of the Basque Country (UPV/EHU), Leioa, Spain; 5 Department of Oncology, University Hospital Puerta del Mar, Cádiz, Spain; 6 Department of Anatomic Pathology, Basurto University Hospital, University of the Basque Country (UPV/EHU), Bilbao, Spain; 7 Department of Surgery, Basurto University Hospital, University of the Basque Country (UPV/EHU), Bilbao, Spain; 8 Department of Cell Biology and Histology, Faculty of Medicine and Nursing, University of the Basque Country (UPV/EHU), Leioa, Spain

**Keywords:** colorectal carcinoma, fibroblast activation protein, cancer associated fibroblast, metastasis

## Abstract

Colorectal cancer (CRC) is a major health problem in elderly people because of its high incidence and high mortality rate. Despite early screening programs, more than half of CRC patients are diagnosed at advanced stages. Fibroblast activation protein-α (FAP) expression in cancer-associated fibroblasts (CAFs) has been associated with a higher risk of metastases and poor survival. Here, we have analyzed the immunohistochemical expression of FAP in 41 adenoma-carcinoma sequences. In addition, FAP expression was analyzed individually and in combination with β-catenin (BCAT), CD44 and Cyclin-D1 expression in primary tumors and in their corresponding lymph node and liver metastases (n=294). Finally, soluble FAP (sFAP) levels in plasma from CRC patients (n=127) were also analyzed by ELISA. FAP was expressed only in CRC tissue and its expression level was found to be higher in tumors exhibiting deeper local invasion and poorer cancer cell differentiation. FAP and concomitant nuclear BCAT expression in cancer cells at the infiltrating front of primary tumors and in lymph node metastases was independently associated with 5- and 10-year cancer specific and disease-free survival. Moreover, lower sFAP levels correlated with poorer survival. These findings support the potential importance of FAP as a biomarker of CRC development and progression.

## INTRODUCTION

Colorectal cancer (CRC) is one of the most common malignancies in terms of incidence and the second leading cause of cancer deaths worldwide [1]. Despite the implementation of early screening programs in several countries, more than half of patients are diagnosed with advanced and metastatic disease (stages III-IV) [2]. Metastasis to liver and lung are the principal causes of death [2, 3]. Therefore, a better understanding of the biomolecular changes underlying colorectal cancer development and metastatic processes is necessary for the design of novel, more effective diagnostic, prognostic and therapeutic tools.

Cancer development and progression rely not only on neoplastic cells themselves, but also on their interaction with other cells of the tumor microenvironment (TME), such as cancer-associated fibroblasts (CAFs) [4, 5]. These cells are the most ubiquitous elements of tumor stroma from epithelial cancers and have been proposed to be the “architects of cancer pathogenesis” [6] because of their participation in the acquisition of several hallmarks of cancer [4, 5].

During the neoplastic transformation of the colorectal epithelium, transforming tumor cells secrete different molecules responsible for fibroblast differentiation into CAFs; the integrity of the basal membrane is disrupted and local invasion of intestinal layers by cancer cells begins [8]. Detecting this early CRC invasion can be diagnostically difficult, both in small biopsy specimens and in adenoma tissues [9].

CAFs can induce tumor invasiveness and disease relapse by promoting the epithelial to mesenchymal transition (EMT) of CRC cells [7, 8], and are among the most influential cells for promoting the generation of cancer stem cell (CSC) subpopulations, which is highly associated with resistance to chemotherapy and radiotherapy [7, 8, 10]. Studies in xenograft models have also shown that CAFs have an important role in the development of pre-metastatic niches, in the nesting of cancer cells in distant organs and in the activation of these cells from their dormant state [8, 11]. Moreover, the activation of CAFs has been associated with the size of CRC metastases in local lymph nodes [12]. For these reasons, the study of the role of CAFs in TME has become a “hot topic” in CRC research [7, 8].

A hallmark of the activation of CAFs is the cell surface expression of fibroblast activation protein-α (FAP), a serine protease with multifunctional properties which is also found in body fluids. This protein has been proposed to be a potential tumor biomarker [13–17], as its expression is more abundant in tumors with invasive phenotypes that are more likely to metastasize [18]. FAP has also been studied as a therapeutic target for CAF-targeted immunotherapies [19]. The relationship between FAP expression in CRC and poor clinical outcome has also been reported [20–22]. However, these studies have been performed using primary tumors and FAP expression in CRC metastases has to date not been characterized [18].

Thus, in this study, we have analyzed the immunohistochemical expression of FAP in a series of colorectal tumors, from adenomatous lesions to advanced primary CRCs, to local and distant metastases. We also analyzed the association between FAP expression in CRC tissues with proliferation, EMT and CSC marker expression such as Cyclin-D1, β-catenin (BCAT) and CD44. Finally, soluble FAP was analyzed by ELISA in plasma samples from CRC patients.

## RESULTS

### FAP expression and the gender and age of CRC patients

The Rho Spearman test was conducted to assess if FAP protein expression varies according to the gender or age of the patients. Results showed no statistically significant differences (p>0.05 in all cases, not shown), allowing us to conclude that the sample has no gender or age bias.

### FAP expression throughout the adenoma-CRC sequence

**Figure 1:**
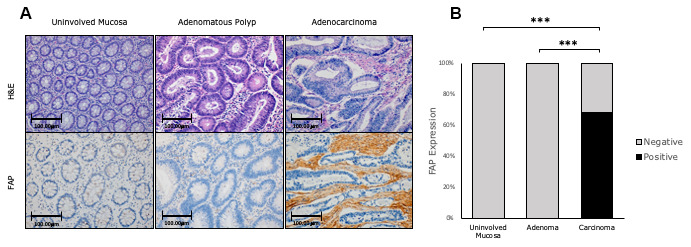
**Immunohistochemical FAP staining along the adenomatous polyp-cancer sequence of CRC.** (**A**) 68,3% of adenocarcinomas were positively stained. Uninvolved colorectal mucosa and adenomas were negative (x200). (**B**) FAP staining was scored as negative or positive. The scores were quantified in each tissue type and statistical significance of the FAP expression pattern among the different tissues was determined by Chi-Square test (*** p<0.001). H&E: Hematoxylin and Eosin. FAP: Fibroblast activation protein-α.

None of the tissues corresponding to uninvolved mucosa or adenomatous polyps showed any FAP protein expression (Chi-square test p<0.001). This protein was exclusively expressed in the stromal fibroblasts present in CRC tissues, where 68.3% of the analyzed CRC cases were classified as positive (Figure 1A, 1B).

### FAP expression and histological subtypes of CRC

**Figure 2:**
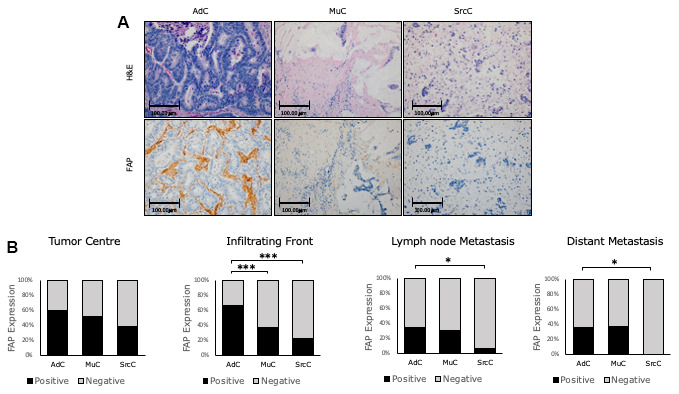
**Immunohistochemical FAP staining according to CRC histologic subtypes.** (**A**) Higher percentage of positive staining was observed in conventional adenocarcinoma (AdC) with respect to mucinous (MuC) and signet ring cell carcinomas (SrcC) in the infiltrating front primary tumour (x200). (**B**) FAP staining intensity was scored as negative or positive. The scores were quantified in each histologic subtype and statistical significance was determined by Chi-Square test (*p<0.05; ***p<0.001). H&E: Hematoxylin and Eosin. FAP: Fibroblast activation protein-α.

We divided the histological subtypes into three groups: conventional adenocarcinoma (AdC), mucinous carcinoma (MuC) and signet ring cell carcinoma (SrcC). FAP expression varied according to histological subtype. Thus, AdCs showed significantly stronger FAP protein expression than MuC and SrcC at the infiltrating front (p<0.001). In local and distant metastases from AdCs, FAP expression was higher than metastases from SrcCs (p<0.05 in both cases) (Figure 2A, 2B).

Taking into consideration such differences, and to avoid bias, all subsequent analyses were conducted only in conventional adenocarcinoma (AdC), which conform the great majority of the cases.

### FAP expression along the conversion of primary tumors into metastases in AdC

**Figure 3:**
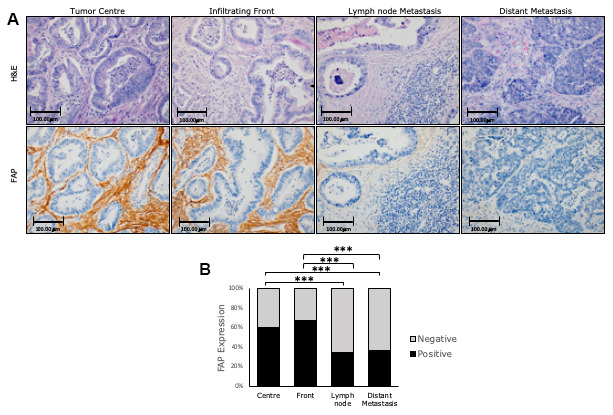
**Immunohistochemical FAP expression in primary (centre and border) and corresponding metastatic (lymph node and liver) tissues of conventional adenocarcinomas (AdCs).** (**A**) Higher percentage of positive staining was observed in primary tumours than in metastases (x200). (**B**) FAP staining intensity was scored as negative or positive. The scores were quantified in each tissue type and statistical significance of FAP expression pattern among the different tissues was determined by Chi-Square test (***p<0.001). H&E: Hematoxylin and Eosin staining. FAP: Fibroblast activation protein-α.

With the aim of exploring the role of FAP protein expression in CRC progression, we measured its expression along the four phases that represent the evolution of CRC into a metastasis, i.e. at the center and infiltrating front of primary tumors, and in local and distant metastases (Figure 3).

In primary tumors, we found overall higher FAP expression at the infiltrating front with respect to the tumor center (Chi-square test p=0.1). When primary and metastatic tissues were compared, FAP expression was significantly lower in both local and distant metastases (p<0.001 in all cases) with respect to the tumor center. However, no significant differences were found between these metastatic tissues (Figure 3A, 3B).

Despite these differences, FAP expression in the primary tumor center and border positively correlated with FAP expression in both lymph nodes (Spearman Rho r=0.166, p=0.028; and r=0.24, p=0.002; respectively) and distant liver metastases (r=0.279, p=0.007; and r=0.256; p=0.016 respectively). Thus, the higher the expression of FAP in primary tumors, the higher the expression of this protein in metastases.

### FAP expression and CRC aggressiveness

In order to study the impact of FAP expression on CRC prognosis, we stratified its expression in primary conventional adenocarcinoma tissues with a range of clinical parameters tightly related to CRC aggressiveness such as histological grade, local invasion (pT), number of affected lymph nodes (N), presence/absence of metastasis (M) and the stage (TNM system).

Results are presented in Table 1. We observed differences between different histological grades, where tissues corresponding to grade 3 (high grade) showed a higher percentage of FAP staining than tissues corresponding to grades 1 and 2 (low grades). These differences were statistically significant at the infiltrating front (p=0.018).

**Table 1:** FAP protein expression pattern according to the different pathological parameters in the center and the infiltrating front of the analyzed primary tumors.

		**Tumor center**				**Tumor front**	
		**Negative staining (%)**	**Positive staining (%)**	**Chi-square (p value)**	**Negative staining (%)**	**Positive staining (%)**	**Chi-square (p value)**
**Grade**	**1-2**	42.3	57.7	0.104	36.1	63.9	**0.018**
	**3**	27.3	72.7		15.2	84.8	
**pT**	**pT1 - pT2**	69.2	30.8	0.003*	30.8	69.2	0.64
	**pT3**	44	56		35.1	64.9	
	**pT4**	25	75		28.6	71.4	
**N**	**N0**	47.2	52.8	0.295	41.7	58.3	0.43
	**N1**	42.6	57.4		29.9	70.1	
	**N2**	33.7	66.3		33.3	66.7	
**M**	**M0**	41.4	58.6	0.644	35.1	64.9	0.432
	**M1**	38.3	61.7		30.1	69.9	
**Stage**	**I-II**	54.2	45.8	0.330	45.8	54.2	0.343
	**III**	35.8	61.5		32.7	67.3	
	**IV**	38.3	61.7		30.1	69.9	

Statistical significance was determined by Chi-square test. Statistically significant values are highlighted in bold.

Regarding local invasion (pT), FAP expression in the tumor center gradually increased as the adenocarcinoma infiltrated the large intestine wall. Thus, FAP expression was significantly higher in tumors invading the visceral peritoneum or other adjacent organs (pT4) than in pT1-2 (p=0.002) and pT3 (p=0.009) adenocarcinomas.

FAP expression at the infiltrating front showed high percentages of positivity in all the pT stages (from 64.9% to 71.4%). In addition, when comparing the center and front from tumors invading the submucosa and muscularis propria (pT1-2), we found FAP expression to be two-fold higher at the infiltrating front (30.8% *vs.* 69.2% positive cases respectively; p=0.05).

When data were stratified according to N, M or stage, no statistically significant differences were observed between the different groups.

### FAP expression in terms of the cancer-specific (CSS) and disease-free survival (DFS) of AdC patients

We aimed to define the role FAP might play in CSS and DFS of CRC affected patients at both 5 and 10 years from diagnosis time. The average follow-up of the AdC series was 45 months ranging from 0 to 184 months.

Results showed no statistically significant relationship between FAP expression from primary and metastatic tissues and patients’ CSS and DFS, neither at 5 nor at 10 years follow-up (Log-rank p>0.05) (Supplementary Table 1A).

### Expression of FAP in combination with BCAT, CD44 and Cyclin-D1 stratified by risk of metastases, CSS and DFS

Data from FAP expression in primary and metastatic tissues were also analyzed in combination with the expression of BCAT, CD44 and Cyclin-D1 (Supplementary Figure 1). Our objectives were: 1) to characterize the association between the combined expression of these biomarkers in primary tumors and local lymph node metastases and the onset of distant metastases; and 2) to evaluate how these double combinations can predict 5- and 10-year CSS and DFS of conventional adenocarcinoma patients.

Multiple logistic regression analyses were performed to carry out the first objective. Significant results were obtained when we studied simultaneous expression of FAP (FAP(+)) in CAFs and β-catenin in the nucleus of CRC cells (BCAT(N)). Table 2 shows that the combined expression of these two biomarkers at the infiltrating front of the primary tumor (as well as pT) was independently associated with metastasis occurrence.

**Table 2:** Predictive model (multiple logistic regression model) according to metastatic status of CRC patients at diagnosis time.

	**Infiltrating front**					
**Multiple logistic regression**	**Variables**	**p value**	**B**	**ExpB**	**Inferior**	**Superior**
	**Grade**	0.745	-0.085	0.919	0.552	1.529
	**pT**	**0.005**	0.793	2.209	1.270	3.842
	**N**	0.349	0.203	1.225	0.801	1.871
	**FAP(+)/BCAT(N)**	**0.026**	0.857	2.355	1.107	5.011
**Final Step of the Wald method**	**pT**	**0.001**	0.865	2.374	1.396	4.039
	**FAP(+)/BCAT(N)**	**0.022**	0.878	2.405	1.132	5.108

Selected independent variables were FAP positive and nuclear BCAT in tumor front, grade, local (pT) and lymph node invasion (N). A stepwise selection procedure (backward Wald method) was used to select the final optimal model. ExpB with confidence interval (CI) is also included. According to the Omnibus test, the model was statistically significant (p=0,002). Hosmer–Lemeshow test (p=0,7). R^2^ Nagelkerke (p=0,1). Statistically significant values are highlighted in bold.

In both the tumor center and in local metastasis, FAP(+)/BCAT(N) was not associated with metastasis occurrence. We also did not find any association between metastasis onset and the expression of both FAP(+)/CD44 high and FAP(+)/Cyclin-D1 high staining in primary tumor and in metastatic lymph node tissues (Supplementary Table 2).

**Figure 4:**
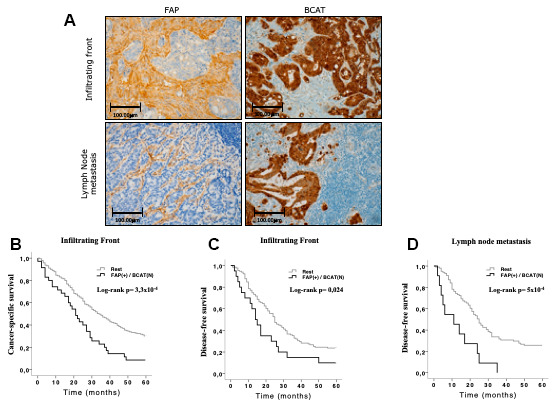
**Immunohistochemical FAP and nuclear BCAT staining in the infiltrating front and in lymph node metastasis.** (**A**) FAP was expressed in CAFs that penetrated within the body of lymphatic nodes. Nuclear and adjacent cytoplasmic BCAT staining represents BCAT signalling translocation from membrane to nucleus (x200). (**B**, **C**) Kaplan-Meier curves and univariate Log-rank test showed that simultaneous expression of FAP in CAFs and nuclear BCAT in AdC cells from the infiltrating front significantly associated with worse 5-year cancer-specific (CSS) and disease-free (DFS) survival of AdC patients. (**D**) The same combination in lymph node metastases was significantly associated to 5-year DFS.

With regard to the second objective, the FAP(+)/BCAT(N) staining combination was associated with worse 5- and 10-year CSS and DFS. Statistically significant results were found at the infiltrating front of the primary tumor for CSS prediction and at the infiltrating front and in local lymph node metastasis for DFS prediction (Figure 4).

The Log-rank test did not reveal any significant association between the other biomarker combinations and patients’ CSS and DFS (Supplementary Table 3).

Additionally, we also performed an individual analysis of BCAT and patients’ survival, in order to ascertain if the combination of FAP and BCAT provides higher predictive value for survival than the individual analysis of each marker. Nuclear expression of BCAT in tumor cells from primary tumor tissues and in metastases was not found to show significant association with the CSS and DFS of AdC patients (Supplementary Table 1B).

Univariate (Supplementary Tables 4 and 5) and multivariate analyses (Table 3) were performed to test whether the FAP(+)/BCAT(N) staining combination at the tumor front and in lymph node metastasis was an independent prognostic factor predicting CSS and DFS. Thus, the Cox regression model showed that this protein combination at the infiltrating front of the primary tumor was an independent prognostic factor for 5-year CSS, together with pT, metastasis and tumor grade. Expression of FAP(+)/BCAT(N) staining combination at the infiltrating front and in lymph node metastasis was also found to be an independent prognostic factor for 5-year FS, together with local invasion (pT) (Table 3). Similar significant results were obtained for the prediction of 10-year CSS and DFS (Supplementary Tables 4, 5 and 6).

**Table 3A:** Predictive model (Cox regression) for 5-year cancer-specific survival (CSS) prediction in AdC patients.

**5-year CSS**	**Variables**	**p value**	**ExpB**	**Inferior**	**Superior**
**Multiple Cox Regression**	**FAP(+)/BCAT(N) tumor front**	**3.5x10^-4^**	1.20	1.09	1.33
	**Grade**	**0.007**	1.49	1.11	1.99
	**pT**	**5.2x10^-5^**	1.89	1.39	2.56
	**N**	0.96	0.99	0.78	1.26
	**M**	**7.3x10^-5^**	1.88	1.38	2.57
**Final Step of Wald Method**	**FAP(+)/BCAT(N) tumor front**	**3.4x10^-4^**	1.20	1.09	1.33
	**Grade**	**0.006**	1.49	1.12	1.97
**pT**	**2.6x10^-5^**	1.88	1.40	2.53
**M**	**7.2x10^-5^**	1.88	1.38	2.57

Selected independent variables were: FAP(+)/BCAT(N) expression in the primary tumor’s infiltrating front, histologic grade, local invasion (pT), lymph node (N) and distant (M) metastases. ExpB with confidence interval (CI) is also included. Statistically significant values are highlighted in bold.

**Table 3B:** Predictive model (Cox regression) for 5-year disease-free survival (DFS) prediction in AdC patients.

		**Tumor front**				**Local metastasis**			
**5-year DFS**	**Variables**	**p value**	**ExpB**	**Inferior**	**Superior**	**p value**	**ExpB**	**Inferior**	**Superior**
**Multiple Cox Regression**	**FAP(+)/BCAT(N)**	**0.011**	1.18	1.04	1.34	**0.002**	1.29	1.09	1.52
	**Grade**	0.174	1.29	0.89	1.86	0.730	1.08	0.70	1.66
	**pT**	**0.018**	1.59	1.08	2.33	**0.009**	1.75	1.15	2.67
	**N**	0.723	1.05	0.79	1.41	0.408	1.21	0.77	1.91
**Final Step of Wald Method**	**FAP(+)/BCAT(N)**	**0.016**	1.17	1.03	1.33	**0.001**	1.31	1.11	1.54
	**pT**	**0.008**	1.65	1.14	2.39	**0.005**	1.80	1.19	2.73

Selected independent variables were: FAP(+)/BCAT(N) expression in the primary tumor’s infiltrating front and in the lymph node metastasis, histologic grade, local invasion (pT), lymph node (N) and distant (M) metastases. ExpB with confidence interval (CI) is also included. Statistically significant values are highlighted in bold.

### Soluble FAP in CRC patient plasma

**Figure 5:**
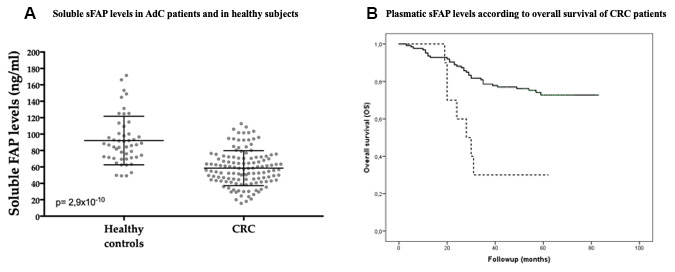
**Soluble FAP (sFAP) plasmatic levels in AdC patients (n=127) and in healthy subjects (n=50).** (**A**) sFAP levels were significantly lower in AdC patients than in controls (T Student, p<0.0001). (**B**) sFAP levels below 30,11 ng/mL were significantly associated with worse overall survival of AdC patients (Log-rank test, p=0.001).

Soluble FAP (sFAP) levels were measured in plasma samples of 127 CRC patients and 50 healthy subjects. Table 4 illustrates the clinical and pathological parameters of the CRC patients. All histological subtypes were conventional adenocarcinomas (AdC). sFAP concentration values followed a normal distribution as indicated by the Kolmogorov-Smirnov test (p>0.05) and there were no age or gender biases affecting sFAP plasma concentration (Pearson test, p>0.05 in both cases). AdC patients had lower sFAP plasma levels (58.6 ng/mL) than control subjects (92.2 ng/mL) (T Student, p<0.0001) (Figure 5A).

**Table 4:** Soluble FAP (sFAP) levels according to clinical and pathological parameters of CRC patients.

**Patients’ clinical and pathological data (n=127)**		**Average (%)**	**sFAP levels (ng/ml)**	**p value**
**Follow-up months (range)**		50 (3-83)		
**Age average (range)**		70 (34-93)		
**Gender**	Male	92 (72%)	57.9	0.61
	Female	35 (28%)	60.1	
**Histologic grade (G)**	G1	6 (5%)	57.4	0,92
	G2	118 (93%)	58.7	
	G3	3 (2%)	53.7	
**Local invasion (pT)**	pT2	32 (25%)	60.3	0.41
	pT3	85 (67%)	58.9	
	pT4	10 (8%)	50.1	
**Affected lymph nodes (N)**	N0	75 (59%)	60.2	0.41
	N1	41 (32%)	57.4	
	N2	11 (9%)	51.4	
**Distant metastases (M)**	M0	123	58.4	0.75
	M1	4	61.9	
**Stage (TNM system)**	I	26 (20%)	56.9	0.29
	II	48 (38%)	62.9	
	III	49 (39%)	54.8	
	IV	4 (3%)	61.9	
**Blood vessel invasion (V)**	No	110 (87%)	58.6	0.97
	Yes	17 (13%)	58.3	
**Lymphatic vessel invasion (L)**	No	103 (81%)	60.6	**0.03**
	Yes	24 (19%)	49.8	
**Perineural invasion (Pn)**	No	108 (85%)	58.5	0.99
	Yes	19 (15%)	58.7	

T Student test and ANOVA test were used for the comparison between two or more than two groups respectively. Significant p values are highlighted in bold.

When sFAP levels were stratified according to several parameters associated with CRC aggressiveness, sFAP levels were found to be significantly lower in patients with lymphatic vessel invasion (L) than the ones without invasion (p<0.05). A similar trend was observed in patients diagnosed with higher grade, pT and pN, but these results were not statistically significant (Table 4). Besides, sFAP levels were inversely correlated with tumor size (Pearson test, r= -0.291, p=0.001).

Cut-off values of sFAP for overall survival analyses were obtained by a Classification and Regression Tree (CRT). A sFAP value of 30,11 ng/mL determined two nodes with significant differences in the percentage of alive patients, 30% *vs* 74% (p=0.03) (Supplementary Figure 2). Kaplan-Meier curves showed that CRC patients with low soluble FAP levels (≤30.11 ng/ml) presented worse overall survival than patients with soluble FAP levels above this cut-off (Log-Rank test p≤0.001) (Figure 5B).

Univariate (Supplementary Table 7) and multivariate Cox regression analyses (Table 5) revealed that plasma sFAP with this cut-off value, histological grade, distant metastasis and vascular invasion are independent prognostic factors for the overall survival (OS) of AdC patients (Table 5).

**Table 5:** Predictive model (Cox regression) for 5-year overall survival (OS) prediction in AdC patients.

**Variables**	**p value**	**B**	**ExpB**	**Inferior**	**Superior**
Grade	**0.044**	1.7	5.475	1.050	28.564
pT	0.355	0.35	1.414	0.678	2.951
N	0.264	0.3	1.353	0.796	2.300
M	0.152	0.99	2.708	0.694	10.574
V	0.100	0.69	2.008	0.875	4.609
Pn	0.261	0.43	1.551	0.721	3.337
Soluble FAP	**0.046**	-1.03	0.356	0.129	0.983
**Final Step of Wald Method**					
Grade	**0.001**	2.52	12.474	2.854	54.527
M	**0.007**	1.67	5.326	1.579	17.969
V	**0.013**	0.97	2.653	1.229	5.728
Soluble FAP	**0.001**	-1.42	0.243	0.104	0.565

Selected independent variables were: soluble FAP, histologic grade, local invasion (pT), lymph node (N) and distant (M) metastases, and vascular (V) and perineural (Pn) invasion. ExpB with confidence interval (CI) is also included. Statistically significant values are highlighted in bold.

## DISCUSSION

The first relevant result of this study was observed in colorectal adenoma-carcinoma sequence. FAP was only expressed by fibroblasts in cancerous tissues, in agreement with the previous work of Henriksson et al. [23]. These authors demonstrated *in vitro* that medium conditioned by CRC cells, but not by adenoma cells, induces FAP expression in CAFs, suggesting that FAP may be a useful diagnostic marker for early CRC invasion [23]. Thus, FAP expression may be specific to CAFs surrounding cancer cells that infiltrate mucosa, but this hypothesis would need to be substantiated with further studies of a series of adenomatous polyps with high grade intraepithelial dysplasia.

FAP expression was lower in CAFs from MuC and SrCC than in conventional adenocarcinoma (AdC). The lower staining could be due to the typical large areas of mucinous degeneration of these tumors that leads to lower collagenous stromal matrix [24], which is the habitat of CAFs. In keeping with this result, it was reported very recently that FAP expression is lower in CRC tumors with low stroma amounts [25]. Therefore, these histotype-related differences in staining patterns should be taken into account for the immunohistochemical evaluation of FAP in CRC tissues.

Tumor border configuration has been proposed as an important histomorphological variable for CRC prognosis [26, 27]. In the margins of the tumor, single CRC cells or clusters with morphological changes of epithelial to mesenchymal transition (EMT) are typical [26, 27]. In addition, host-related factors also influence the invasive behavior of these margins and, therefore, the prognosis of CRC patients [25, 28].

From a molecular perspective, the translocation of BCAT to the nucleus is a key event which confers invasive properties on CRC cells. There, it activates target genes that encode markers such as CD44 and cyclin-D1, inducing EMT, CSC-like phenotype and cell proliferation [29, 30]. These markers have been found to be associated with a poorer prognosis of CRC patients [30–31]. In addition, functional studies have demonstrated that FAP expression in the membrane of CAFs induces BCAT-related pathway phenomena in CRC cells [7, 33], which can explain in part the impact of FAP in CRC development and prognosis [20–22].

Taking all this evidence into account, we decided to analyze FAP expression individually and in combination with these markers in the tumor center and at the infiltrating front of primary AdCs. Thus, the tumor front showed high FAP expression in high histological grade tumors and in all the stages of local invasion (pT). Furthermore, FAP positivity in CAFs and concomitant nuclear expression of BCAT in neoplastic cells from the tumor margins were independently associated with a higher risk of metastasis and with worse CSS and DFS of conventional adenocarcinoma patients. These results suggest that the impact of FAP on the aggressive behavior of primary AdCs takes place mainly at the infiltrating front, and that this expression may be associated with BCAT-related phenomena in tumor cells [7, 33].

These findings also illustrate the importance of the combined analyses of immunohistochemical results, which can yield relevant information not revealed by individual analyses. In this regard, Herrera et al. [22] demonstrated that the combination of M2 macrophage and CAF markers such as FAP identifies groups of advanced CRC patients with significant differences in the progression of the disease.

We observed significant differences in FAP expression between primary tumors and their corresponding local and distant metastases. Nevertheless, there was a positive correlation between FAP expression in both locations, reminiscent of our previous findings in renal cancer [34]. FAP was found to be expressed in CAFs that penetrated into the body of lymph nodes, which is a sign of fibroblast activation associated with cancer cell colonization [12]. Moreover, concomitant FAP/nuclear BCAT expression in lymph node metastases was independently associated with worse DFS of AdC patients.

It is known that CAFs from primary and metastatic sites can have different origins and phenotypes [11]. However, increasing evidence has shown that CAFs also circulate together with cancer cells, growth factors and exosomes from the primary site to distant organs during the metastatic process [35, 36]. This close communication between primary and secondary sites influences the progression of metastasis and the outcome of patients [11, 35–37]. Further studies will be necessary to clarify if the present findings are associated with the coordinated interaction of primary and distant sites or whether they represent independent phenomena.

FAP has been mainly described in activated fibroblasts from cancer, chronic inflammatory and fibrotic lesions [17, 18]. Thus, higher sFAP levels would be expected in cancer patients in comparison with healthy subjects. However, we found that plasma samples from conventional adenocarcinoma patients had lower levels of the soluble fraction of FAP (sFAP) than those found in control subjects. Furthermore, lower sFAP levels were found to correlate with higher tumor size and invasion of lymphatic vessels, and independently associated with worse overall survival. A number of studies in several cancers showed similarly surprisingly reduced levels of sFAP associated with the cancer state [14–16]. Higher levels in healthy subjects indicates that sFAP may not be produced by the tumor tissues. Indeed, it has recently been reported that skeletal muscle, liver and bone marrow may be physiological sources of sFAP [16]. Lower sFAP levels in pathological conditions may reflect a systemic reaction to the presence of a developing tumor, a phenomenon comparable to decreased plasma level of negative acute-phase proteins seen in inflammatory processes [15, 16, 38]. Similarly, paradoxical results have also been reported for other peptidases in CRC [39, 40], underlining the need for further analyses, since it will be vital to ascertain the origin of plasma peptidases before they can be considered to be reliable biomarkers for liquid biopsies of CRC patients.

In summary, the present findings corroborate the potential usefulness of FAP as a biomarker of CRC development and progression. Further studies will be necessary to contribute to a more detailed understanding of the role of FAP in cross-communications between cells of TME from primary and metastatic tumors. Since CRC represents a major health problem in developed countries [1], the importance of such studies which will contribute to the design of more effective diagnostic, prognostic and therapeutic tools, cannot be underestimated.

## MATERIALS AND METHODS

The authors declare that all the experiments carried out in this study comply with the current Spanish and European Union legal regulations. Samples and data from patients included in this study were provided by the Basque Biobank for Research-OEHUN (https://www.biobancovasco.org). All patients were informed about the potential use for research of their surgically resected tissues, and manifested their consent by signing a specific document approved by the Ethical and Scientific Committees of the Basque Country Public Health System (Osakidetza) (CEIC 11/51 and CEIC 16/108).

### Patients

Three groups of samples were used in this research: 1) primary (n=294) and metastatic [(lymph node, n=227) and hepatic metastases, n=119)] tissues from 294 patients diagnosed with advanced CRC were immunohistochemically analyzed; Table 6 summarizes the clinical and pathological characteristics of these patients; 2) a series of normal colonic mucosa, adenomas with low grade dysplasia and adenocarcinomas from the same patients (n=41) was used to immunohistochemically analyze the polyp-CRC sequence; and 3) plasma samples from 127 CRC patients and from 50 healthy subjects (see characteristics in Table 4) were used for the determination of the concentration of the soluble fraction of FAP.

**Table 6:** Clinical and pathological parameters of CRC patients for the immunohistochemical analysis of FAP protein expression.

**Patients’ clinical and pathological data (n=294)**		**Average (%)**
**Age average (range)**		70 (29-93)
**Follow-up months (range)**		44 (0-188)
**Gender**	Male	203 (69%)
	Female	91 (31%)
**Histologic subtype**	Conventional Adenocarcinoma (AdC)	231 (79%)
	Mucinous carcinoma (MuC)	50 (17%)
	Signet ring cell carcinoma (SrcC)	13 (4%)
**Histologic grade (G)**	G1	37 (13%)
	G2	161 (55%)
	G3	95 (32%)
**Local invasion (pT)**	pT1	2 (<1%)
	pT2	13 (4%)
	pT3	178 (61%)
	pT4	101 (34%)
**Affected lymph nodes (N)**	N0	47 (16%)
	N1	130 (44%)
	N2	117 (40%)
**Distant metastases (M)**	M0	182 (62%)
	M1	112 (38%)
Stage (TNM system)*	I	7 (2%)
	II	25 (9%)
	III	150 (51%)
	IV	112 (38%)

### Immunohistochemistry

Formalin-fixed and paraffin-embedded tissues were immunostained with antibodies specific for FAP (1:70 dilution, Ab53066 Abcam), BCAT (Ready to use, 760-4242, Roche-Ventana), CD44 (Ready to use, 790-4537, Roche-Ventana) and Cyclin-D1 (Ready to use, sc-718, Santa Cruz biotechnology).

The immunostaining process was performed following routine methods in an automatic immunostainer (DakoAutostainer Plus, Dako-Agilent). Briefly, antigen retrieval was carried out in low pH buffer (K8005, Dako) for 20 minutes at 95ºC. The samples were incubated with the primary antibody for 50 minutes at room temperature. Then, the primary antibody was washed and samples were incubated for 20 minutes with secondary anti-rabbit antibody (K8021, Dako). The EnVision-Flex detection system together with a HRP enzyme labelled polymer (SM802, Dako) was used. The presence of bound HRP-labeled secondary antibody was visualized with diaminobenzidine (DAB) solution (DM827, Dako) followed by counterstaining with hematoxylin (K8008, Dako).

For staining evaluation, slides were reviewed under light microscopy. For FAP evaluation stromal staining was scored as negative, +, ++ and +++ according to the semi-quantitative scale described by Henry et al [20]. Negative/ + and ++/+++ levels were grouped in order to compare no/weak (≤ 10% stromal cells) with moderate/high (>10% stromal cells) staining groups [20]. Membranous staining of BCAT was interpreted as normal, and nuclear staining was considered translocational expression [41]. CD44 and Cyclin-D1 staining was scored as negative, +, ++ and +++ attending not only to expression in neoplastic epithelial cells but also to non-immune stromal cells. For statistical purposes two groups of cases were defined in terms of negative/ + (low) and ++/+++ (high) expression levels [42–45].

The specimens were independently evaluated by two observers and discordant cases were jointly reviewed followed by a conclusive judgment.

### ELISA assays

In order to determine the levels of soluble FAP, the sFAPDuoSet ELISA kit (R&D Systems, DY3715) was used [46, 47]. 100 μl of standards, reagent blank and plasma samples (1/100 dilution) were plated into a 96-well plate and incubated overnight at 4ºC. Wells were washed 4 times and 100 μl of labelled FAP antibody was added (except to the blank) and incubated for 1 hour at 4ºC. Then, wells were washed 5 times and 100 μl of chromogen was added and incubated for 30 minutes at room temperature. 100ul of stop solution was added to each well and absorbance was measured at 450nm against reagent blank.

### Statistical analysis

SPSS® 24.0 software was used for the statistical analysis.

A Kolmogorov-Smirnov test was used to determine whether data obtained from tissue and plasma samples followed or not a normal distribution. Based on this information, data were analyzed with parametric or non-parametric tests.

We performed Pearson and Spearman Rho tests to evaluate the correlation between FAP expression and patient age and gender, and to correlate FAP expression from primary tumors and metastases. T-Student and ANOVA tests were used to compare plasmatic FAP levels between two groups or more (respectively). Chi-square (χ2) test was used to analyze the categorical FAP expression (negative/positive) throughout the adenoma-CRC sequence, and the association with tissue FAP expression depending on pathological variables.

To perform cancer-specific survival (CSS) and disease-free survival (DFS) analyses, groups were created by cut-off points following different methods: for plasma analysis of FAP, a classification and regression tree (CRT) method was employed. For tissue analyses, cut-off points were based on categorical expression of FAP (negative *vs.* positive). We also created groups combining data from FAP expression with β-catenin, CD44 and cyclin-D1 expression in CRC tissues. The following categorical groups of two markers were created (see Supplementary Figure 1): 1) FAP positive and nuclear β-catenin *vs.* rest of combination options between these two markers; 2) FAP positive and high CD44 *vs.* the rest, and 3) FAP positive and high Cyclin-D1 *vs.* the rest.

Kaplan-Meier curves and log-rank tests were performed to evaluate the association between the above-described groups and the CSS and DFS of CRC patients. Finally, multivariate analyses were used to test the independent effects of FAP expression (alone or combined with three other markers) and clinical and pathological variables on CSS and DFS (by Cox regression model) and on the onset of metastasis (by multiple logistic regression).

## Supplementary Material

Supplementary Figures

Supplementary Tables
