# Supplementary material for: Altered expression of fibroblast activation protein-α (FAP) in colorectal adenoma-carcinoma sequence and in lymph node and liver metastases
Source: Aging (Albany NY). 2020 May 19;12(11):10337–58. doi: 10.18632/aging.103261 (PMC7346028; doi:10.18632/aging.103261)
Supplement: Supplementary Figures [file aging-12-103261-s002..pdf]

# SUPPLEMENTARY FIGURES

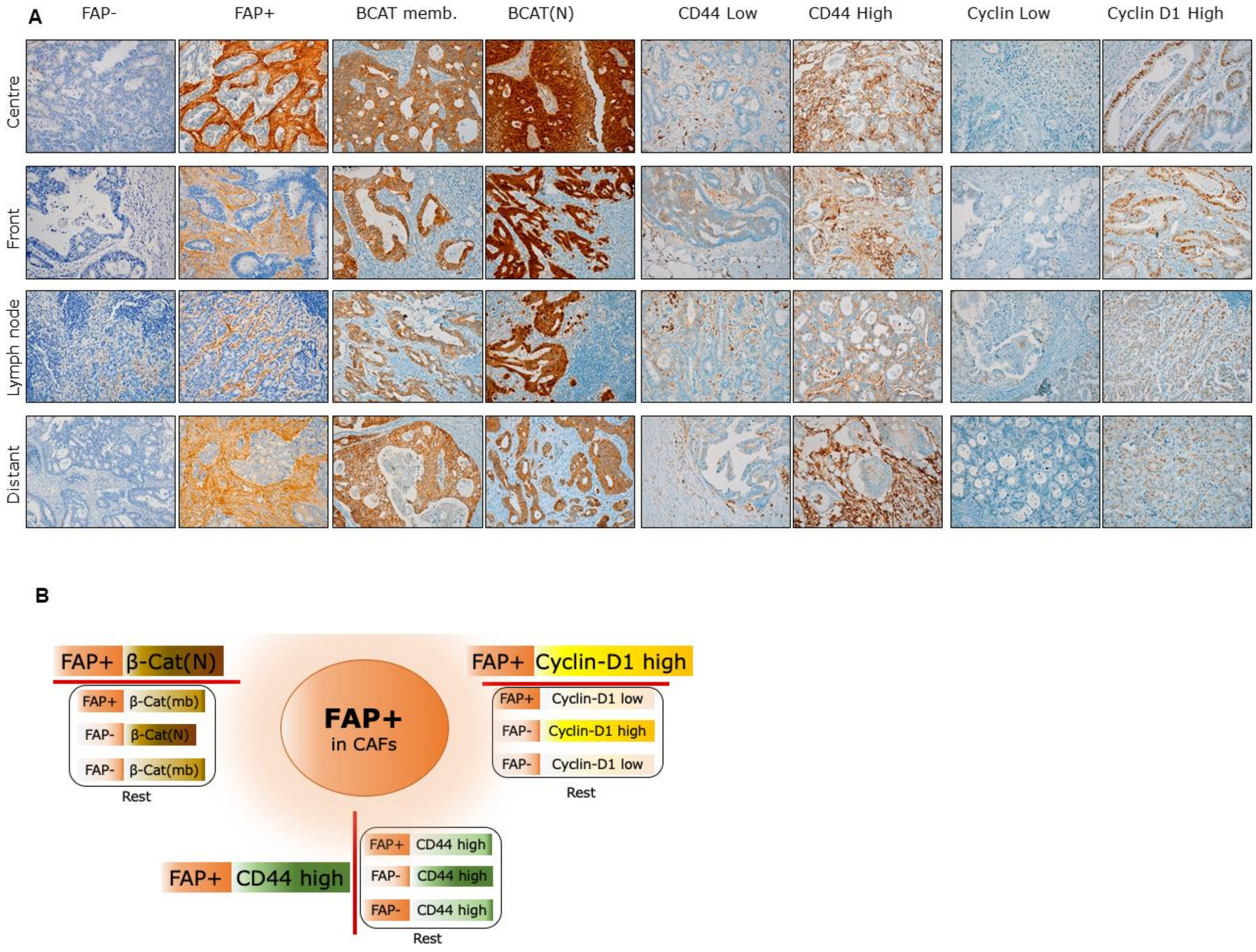


**Supplementary Figure 1.** (**A**) Images of immunohistochemical stains of FAP, BCAT, CD44 and Cyclin-D1. It is illustrated the entire spectrum of immunohistochemical results obtained in the different anatomical locations studied throughout the evolution AdC (primary tumour centre, infiltrative front, lymph node metastasis and distant liver metastasis). (**B**) Different combinations of TME immunohistochemical markers. The relationship between FAP+ / nuclear BCAT, FAP+ / CD44 high and FAP+ / Cyclin D1 high regarding to the rest of combinations in each pair of markers was studied. Data from tumour centre, infiltrating front and lymph node metastases were combined.

**Classification and Regression Tree (CRT) for soluble FAP and CRC patients' survival**

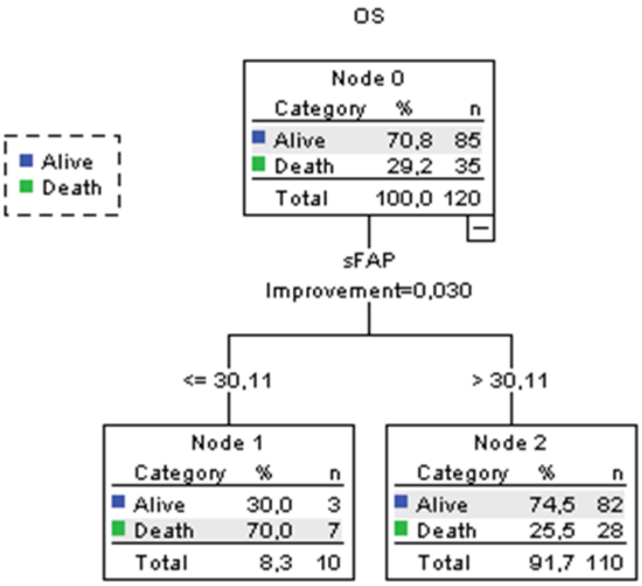


**Supplementary Figure 2. Classification and Regression Tree (CRT).** A sFAP value of 30,11 ng/mL determined two nodes with significant differences in the percentage of alive patients (p=0.03).
