# Supplementary material for: Altered expression of fibroblast activation protein-α (FAP) in colorectal adenoma-carcinoma sequence and in lymph node and liver metastases
Source: Aging (Albany NY). 2020 May 19;12(11):10337–58. doi: 10.18632/aging.103261 (PMC7346028; doi:10.18632/aging.103261)
Supplement: Supplementary Tables [file aging-12-103261-s001..pdf]

SUPPLEMENTARY TABLES

Supplementary Table 1A. Log-rank test results of the association between FAP expression in primary tumors and metastases and 5- and 10-year CSS and DFS of AdC patients.

FAP (Location)	5-year CSS (p value)	10-year CSS (p value)	5-year DFS (p value)	10-year DFS (p value)
Tumor Center	0.71	0.94	0.91	0.93
Infiltrating front	0.46	0.32	0.72	0.71
Local metastasis	0.86	0.88	0.25	0.22
Distant metastasis	0.49	0.45	0.56	0.67

Supplementary Table 1B. Log-rank test results of the association between BCAT expression (membrane/cytoplasmic *vs* nuclear) in primary tumors and metastases and 5- and 10-year CSS and DFS of AdC patients.

FAP (Location)	5-year CSS (p value)	10-year CSS (p value)	5-year DFS (p value)	10-year DFS (p value)
Tumor Center	0.93	0.69	0.6	0.49
Infiltrating front	0.08	0.45	0.08	0.24
Local metastasis	0.37	0.9	0.71	0.81
Distant metastasis	0.21	0.33	0.58	0.52

Supplementary Table 2A. Multiple Logistic Regression Model according to metastatic status of CRC patients at diagnosis time: FAP positive and nuclear BCAT in tumor center.

	Tumor center					
	Variables	p value	B	ExpB	Inf	Sup
Multiple logistic regression	Grade	0.58	-0.14	0.86	0.52	1.45
	pT	**0.001**	0.92	2.5	1.43	4.37
	N	0.3	0.23	1.25	0.82	1.92
	FAP(+)/BCAT(N)	0.24	-0.14	0.87	0.69	1.09

ExpB and confidence interval (CI) is included. According to the Omnibus test, the model was statistically significant (p=0.003). Hosmer–Lemersow test (p=0.66). R^2 Nagelkerke (p=0.09). Statistically significant values are highlighted in bold.

Supplementary Table 2B. Multiple Logistic Regression Model according to metastatic status of CRC patients at diagnosis time: FAP positive and nuclear BCAT in lymph node local metastasis.

	Local metastasis					
	Variables	p value	B	ExpB	Inferior	Superior
Multiple logistic regression	Grade	0.79	-0.07	0.93	0.53	1.62
	pT	**0.04**	0.65	1.92	1.04	3.54
	N	0.16	0.44	1.55	0.84	2.87
	FAP(+)/CD44	0.45	-0.11	0.89	0.66	1.2

ExpB and confidence interval (CI) is included. According to the Omnibus test, the model was not statistically significant (p=0.08). Hosmer–Lemersow test (p=0.09). R^2 Nagelkerke (p=0.06). Statistically significant values are highlighted in bold.

Supplementary Table 2C. Multiple Logistic Regression Model according to metastatic status of CRC patients at diagnosis time: FAP positive and high staining of CD44 in tumor center.

	Tumor Center					
	**Variables**	**p value**	**B**	**ExpB**	**Inferior**	**Superior**
**Multiple logistic regression**	**Grade**	0.38	-0.22	0.79	0.48	1.33
	**pT**	**0.002**	0.87	2.38	1.38	4.12
	**N**	0.26	0.24	1.27	0.84	1.95
	**FAP(+)/CD44**	0.46	-0.57	0.94	0.81	1.09

ExpB and confidence interval (CI) is included. According to the Omnibus test, the model was statistically significant (p=0.004). Hosmer–Lemersow test (p=0.82). R^2 Nagelkerke (p=0.09). Statistically significant values are highlighted in bold.

Supplementary Table 2D. Multiple Logistic Regression Model according to metastatic status of CRC patients at diagnosis time: FAP positive and high staining of CD44 in the infiltration front.

	Infiltrating Front					
	**Variables**	**p value**	**B**	**ExpB**	**Inferior**	**Superior**
**Multiple logistic regression**	**Grade**	0.54	-0.16	0.85	0.5	1.44
	**pT**	**0.001**	0.96	2.62	1.5	4.57
	**N**	0.48	0.15	1.17	0.76	1.79
	**FAP(+)/CD44**	0.24	0.11	1.11	0.93	1.33

ExpB and confidence interval (CI) is included. According to the Omnibus test, the model was statistically significant (p=0.003). Hosmer–Lemersow test (p=0.89). R^2 Nagelkerke (p=0.1). Statistically significant values are highlighted in bold.

Supplementary Table 2E. Multiple Logistic Regression Model according to metastatic status of CRC patients at diagnosis time: FAP positive and high staining of CD44 in local lymph node metastasis.

	Local metastasis					
	**Variables**	**p value**	**B**	**ExpB**	**Inferior**	**Superior**
**Multiple logistic regression**	**Grade**	0.54	-0.17	0.84	0.48	1.46
	**pT**	0.06	0.57	1.77	0.97	3.23
	**N**	0.18	0.41	1.51	0.82	2.78
	**FAP(+)/CD44**	0.97	0.01	1.01	0.8	1.25

ExpB and confidence interval (CI) is included. According to the Omnibus test, the model was statistically significant (p=0.003). Hosmer–Lemershow test (p=0.89). R^2 Nagelkerke (p=0.1).

Supplementary Table 2F. Multiple Logistic Regression Model according to metastatic status of CRC patients at diagnosis time: FAP positive and high staining of Cyclin-D1 in the tumor center.

	Tumor center					
	**Variables**	**p value**	**B**	**ExpB**	**Inferior**	**Superior**
**Multiple logistic regression**	**Grade**	0.39	-0.22	0.80	0.48	1.33
	**pT**	**0.002**	0.86	2.37	1.37	4.11
	**N**	0.22	0.26	1.30	0.85	1.99
	**FAP(+)/Cyclin-D1**	0.27	0.08	1.09	0.94	1.26

ExpB and confidence interval (CI) is included. According to the Omnibus test, the model was statistically significant (p=0.002). Hosmer–Lemersow test (p=0.61). R^2 Nagelkerke (p=0.09). Statistically significant values are highlighted in bold.

Supplementary Table 2G. Multiple Logistic Regression Model according to metastatic status of CRC patients at diagnosis time: FAP positive and high staining of Cyclin-D1 in the infiltration front.

	Infiltrating front					
	**Variables**	**p value**	**B**	**OR**	**Inferior**	**Superior**
**Multiple logistic regression**	**Grade**	0.425	-0.21	0.81	0.49	1.35
	**pT**	**0.003**	0.83	2.29	1.33	3.96
	**N**	0.223	0.27	1.31	0.85	2.02
	**FAP(+)/Cyclin-D1**	0.261	0.08	1.09	0.94	1.26

ExpB and confidence interval (CI) is included. According to the Omnibus test, the model was statistically significant (p=0.005). Hosmer–Lemersow test (p=0.96). R^2 Nagelkerke (p=0.09). Statistically significant values are highlighted in bold.

Supplementary Table 2H. Multiple Logistic Regression Model according to metastatic status of CRC patients at diagnosis time: FAP positive and high staining of Cyclin-D1 in local lymph node metastasis.

	Local metastasis					
	**Variables**	**p value**	**B**	**ExpB**	**Inferior**	**Superior**
**Multiple logistic regression**	**Grade**	0.75	-0.08	0.92	0.54	1.57
	**pT**	0.07	0.54	1.72	0.96	3.10
	**N**	0.36	0.28	1.32	0.73	2.41
	**FAP(+)/Cyclin-D1**	0.28	0.11	1.12	0.91	1.36

ExpB and confidence interval (CI) is included. According to the Omnibus test, the model was not statistically significant (p=0.138). Hosmer–Lemersow test (p=0.5). R^2 Nagelkerke (p=0.05).

Supplementary Table 3. Log-rank test results of the association between FAP and BCAT, CD44 and Cyclin-D1, and cancer-specific (CSS) and disease-free survival (DFS) of AdC patients.

**FAP(+)/BCAT(N) (Location)**	**5-year CSS (p value)**	**10-year CSS (p value)**	**5-year DFS (p value)**	**10-year DFS (p value)**
Tumor Center	0.94	0.54	0.86	0.82
Infiltrating front	**$3.3x10^{-4}$**	**0.001**	**0.024**	**0.033**
Local metastasis	0.07	0.07	**$5x10^{-4}$**	**$5x10^{-4}$**
Distant metastasis	0.98	0.5	0.86	0.95
**FAP(+)/CD44 high**				
Tumor Center	0.72	0.98	0.71	0.95
Infiltrating front	0.34	0.47	0.22	0.46
Local metastasis	0.39	0.17	0.06	0.06
Distant metastasis	0.97	0.76	0.63	0.72
**FAP(+)/CyclinD1 high**				
Tumor Center	0.5	0.83	0.84	0.81
Infiltrating front	0.71	0.46	0.37	0.78
Local metastasis	0.22	0.15	0.15	0.09
Distant metastasis	0.29	0.11	0.49	0.5

Supplementary Table 4A. Univariate analysis (Cox regression model) of clinical and pathological variables and FAP(+)/BCAT(N) expression in tumor front: analysis of 5-year CSS of AdC patients.

Variables	p value	ExpB	Inferior	Superior
**Grade**	**0.012**	1.441	1.085	1.915
**pT**	**$1x10^{-6}$**	2.021	1.528	2.673
**N**	**0.022**	1.284	1.036	1.592
**M**	**$1x10^{-6}$**	2.161	1.596	2.927
**FAP(+) / BCAT(N) Tumor front**	**$4.9x10^{-4}$**	1.189	1.079	1.311

ExpB and confidence interval (CI) is included. Statistically significant values are highlighted in bold.

Supplementary Table 4B. Univariate analysis (Cox regression model) of clinical and pathological variables and FAP(+)/BCAT(N) expression in tumor front: analysis of 10-year CSS of AdC patients.

Variables	p value	ExpB	Inferior	Superior
**Grade**	0.15	1.2	0.94	1.55
**pT**	**$8x10^{-6}$**	1.78	1.38	2.29
**N**	0.15	1.15	0.95	1.38
**M**	**$4x10^{-6}$**	1.9	1.44	2.49
**FAP(+) / BCAT(N) Tumor front**	**0.002**	1.16	1.06	1.27

ExpB and confidence interval (CI) is included. Statistically significant values are highlighted in bold.

Supplementary Table 5A. Univariate analysis (Cox regression model) of clinical and pathological variables and FAP(+)/BCAT(N) expression in tumor front and local lymph node metastasis: analysis of 5-year disease-free survival (DFS) of AdC patients.

Variables	p value	ExpB	Inferior	Superior
**Grade**	0.10	1.33	0.94	1.87
**pT**	**0.004**	1.68	1.17	2.39
**N**	0.09	1.25	0.96	1.62
**FAP(+) / BCAT(N) Tumor front**	**0.028**	1.15	1.01	1.31
**FAP(+) / BCAT(N) Local metastasis**	**0.001**	1.31	1.11	1.54

ExpB with confidence interval (CI) is included. Statistically significant values are highlighted in bold.

Supplementary Table 5B. Univariate analysis (Cox regression model) of clinical and pathological variables and FAP(+)/BCAT(N) expression in tumor front and local lymph node metastasis: analysis of 10-year disease-free survival (DFS) of AdC patients.

Variables	p value	ExpB	Inferior	Superior
**Grade**	0.38	1.15	0.84	1.57
**pT**	**0.02**	1.48	1.06	2.07
**N**	0.29	1.13	0.89	1.44
**FAP(+) / BCAT(N) Tumor front**	**0.04**	1.13	1	1.28
**FAP(+) / BCAT(N) Local metastasis**	**0.001**	1.31	1.11	1.54

ExpB with confidence interval (CI) is included. Statistically significant values are highlighted in bold.

Supplementary Table 6A. Predictive model (Cox regression) for 10-year cancer-specific survival (CSS) prediction in AdC patients.

		Tumor front			
**10-year CSS**	**Variables**	**p value**	**ExpB**	**Inferior**	**Superior**
**Multiple Cox Regression**	**pT**	**$1x10^{-4}$**	1.68	1.28	2.19
	**M**	**0.001**	1.64	1.24	2.17
	**FAP(+)/BCAT(N)**	**0.003**	1.15	1.05	1.26

Selected independent variables were: FAP(+)/BCAT(N) expression in the primary tumor's infiltrating front, local invasion (pT) and distant (M) metastases. ExpB with confidence interval (CI) is also included. Statistically significant values are highlighted in bold.

Supplementary Table 6B. Predictive model (Cox regression) for 10-year disease-free survival (DFS) prediction in AdC patients.

		Tumor front				Local metastasis			
**10-year DFS**	**Variables**	**p value**	**ExpB**	**Inferior**	**Superior**	**p value**	**ExpB**	**Inferior**	**Superior**
**Multiple Cox**	**pT**	**0.03**	1.47	1.04	2.08	0.02	1.61	1.09	2.39
**Regression**	**FAP(+)/BCAT(N)**	**0.03**	1.14	1.01	1.29	0.01	1.31	1.11	1.54

Selected independent variables were: FAP(+)/BCAT(N) expression in the primary tumor's infiltrating front and in lymph node metastasis, and local invasion (pT). ExpB with confidence interval (CI) is also included. Statistically significant values are highlighted in bold.

Supplementary Table 7. Univariate analysis (Cox regression model) of clinical and pathological variables and plasmatic FAP levels for AdC patients' 5-year overall survival prediction.

Variables	p value	B	ExpB	Inferior	Superior
**Grade**	**$2.7x10^{-4}$**	2.69	14.77	3.46	63.08
**pT**	**$1.6x10^{-4}$**	1.17	3.23	1.76	5.95
**N**	**0.001**	0.76	2.13	1.38	3.28
**M**	**0.016**	1.46	4.31	1.31	14.13
**V**	**$3.4x10^{-4}$**	1.32	3.73	1.82	7.67
**L**	**0.203**	0.48	1.62	0.77	3.41
**Pn**	**$3.9x10^{-4}$**	1.23	3.43	1.74	6.79
**Soluble FAP**	**0.001**	-1.34	0.26	0.11	0.59

ExpB with confidence interval (CI) is also included. Statistically significant values are highlighted in bold
